# Supplementary material for: A Practical and Analytical Comparative Study of Gel-Based Top-Down and Gel-Free Bottom-Up Proteomics Including Unbiased Proteoform Detection
Source: Cells. 2023 Feb 26;12(5):747. doi: 10.3390/cells12050747 (PMC10000902; doi:10.3390/cells12050747)
Supplement: Supplementary file 1 [file cells-12-00747-s001.zip › Supplementary Materials Ercan et al.pdf]

cells

Supplementary Materials: A practical and analytical comparative study of gel-based top-down and gel-free bottom-up proteomics including unbiased proteoform detection

Huriye Ercan, Ulrike Resch, Felicia Hsu, Goran Mitulovic, Andrea Bileck, Christopher Gerner, Jae-Won Yang, Margarethe Geiger, Ingrid Miller and Maria Zellner

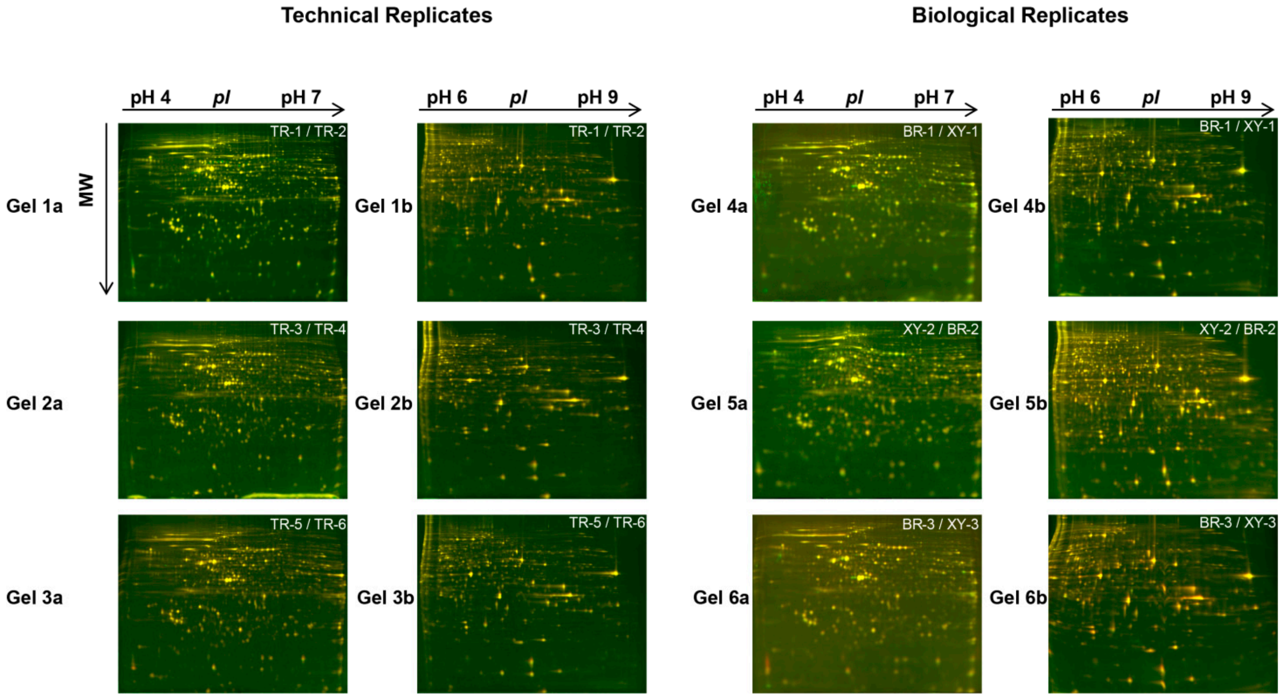

Figure S1. Overview of 2D-DIGE gels from technical and biological replicates in the pH range 4-7 and 6-9. The green spots indicate Cy3-labeled samples, while the red spots indicate Cy5-labeled samples. Yellow spots indicate that the protein spots are equal abundant in both the Cy3- and Cy5- labeled samples. Abbreviations: 2D-DIGE – two-dimensional differential in-gel electrophoresis; MW – molecular weight; pI – isoelectric point; TR – technical replicate; BR – biological replicate.

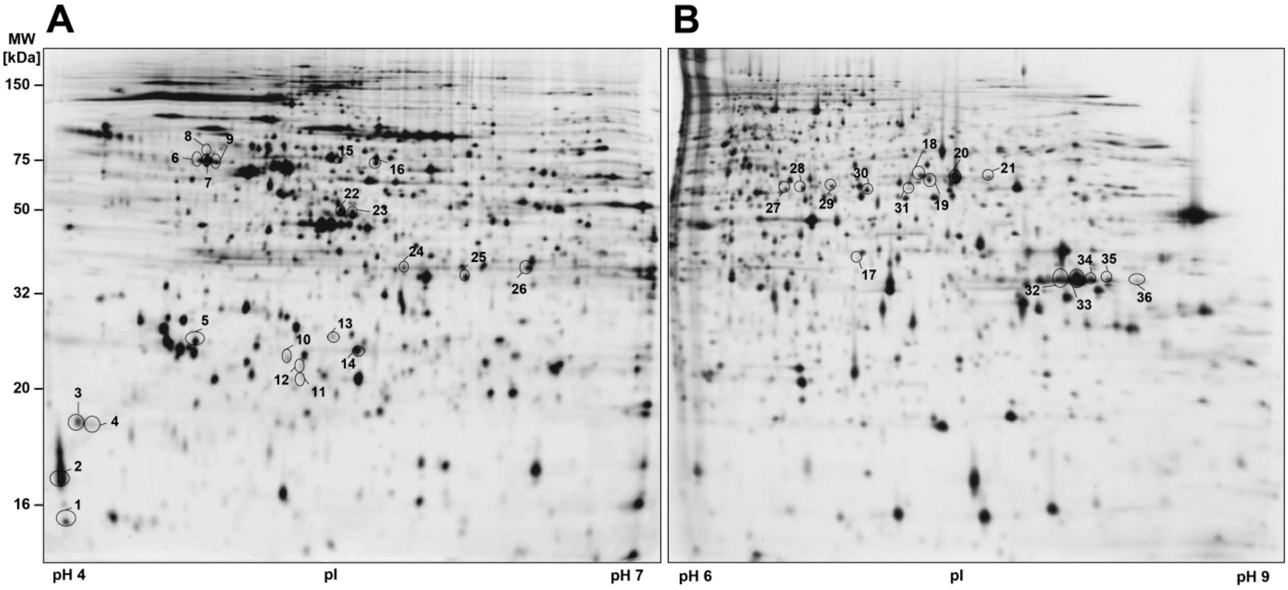

Figure S2. Representative image of a 2D-DIGE gel of human prostate cancer cell line DU145 at pH 4-7 **(A)** and pH 6-9 **(B)**. Circled spots are found in both proteomics methods, 2D-DIGE and label-free shotgun (see Table 1 for more details).

A

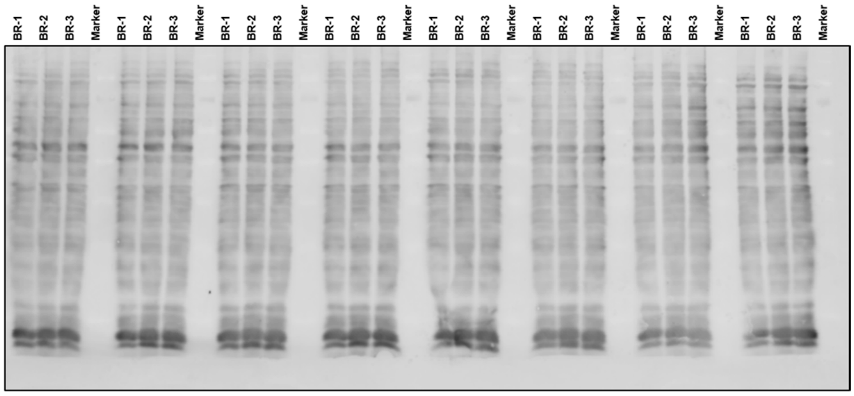

B

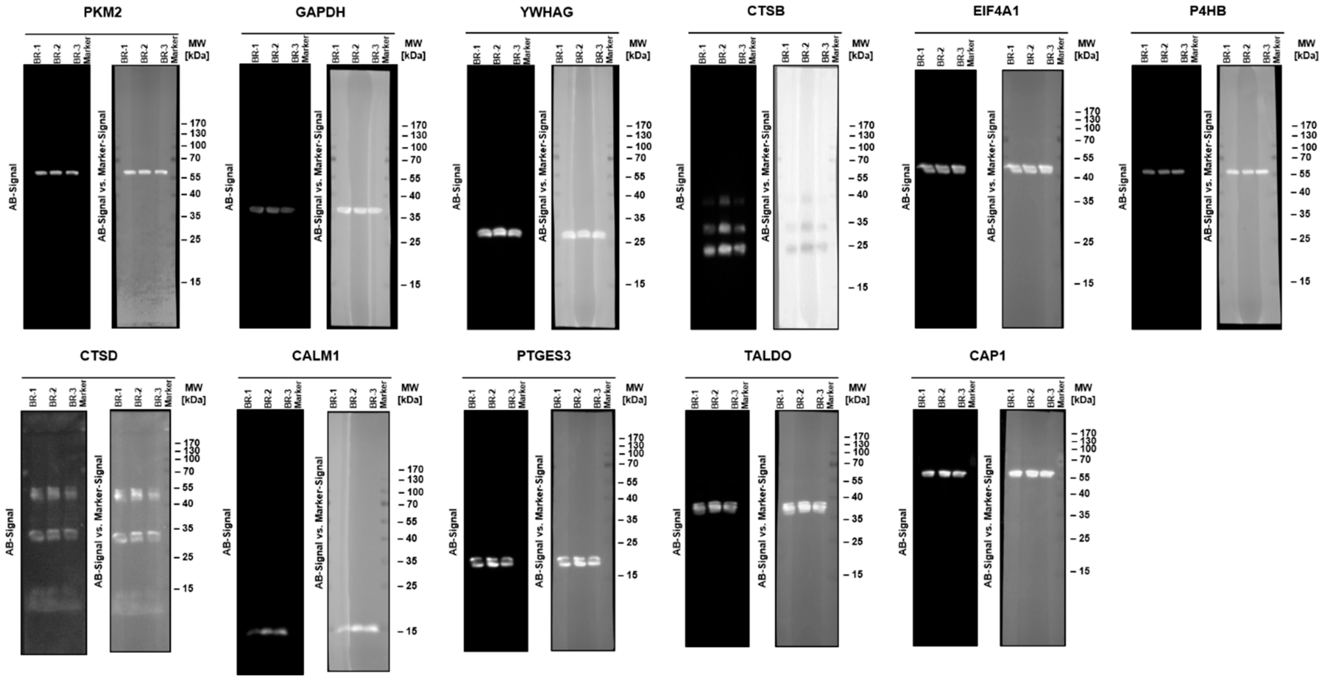

Figure S3. Validation of PKM2, GAPDH, YWHAG, CTSB, EIF4A1, P4HB, CTSD, CALM1, PTGES3, TALDO and CAP1 antibodies by one-dimensional Western blot analysis. **(A)** Ruthenium-based whole-protein stain of blotted proteins. Twelve µg of DU145 protein lysate was loaded per lane on an 11.5% SDS-PAGE with 28 sample wells and separated according to their molecular weight. The three biological DU145 replicates (BR-1, BR-2 and BR-3) of the previous proteomics study were alternately applied to both 1D gels with a pre-stained protein molecular weight marker in the MW range of 170 kDa - 15 kDa, and afterwards blotted onto two PVDF membranes. The apparently empty lanes contain this marker, which is visible to the naked eye but not with the device setting for the ruthenium stain. With visual guidance of the lanes with the separated protein molecular weight markers, the membranes were cut into 11 pieces, each containing the three biological DU145 replicates and one sample of the protein molecular weight marker. **(B)** These PVDF membranes were stained with antibodies against PKM2, GAPDH, YWHAG, CTSB, EIF4A1, P4HB, CTSB, CALM1, PTGES3, TALDO and CAP1, respectively. The specific signals were visualized by staining with the respective secondary HRP-conjugated antibody and with a substrate for chemiluminescence (left image of the respective protein). The signals were detected with the respective wavelength and filter settings of a UVP ChemStudio Imager. To check the correctness of the respective molecular weights of the different proteins detected, an overlay was made with the protein molecular weight marker (right picture of the respective Western blot).

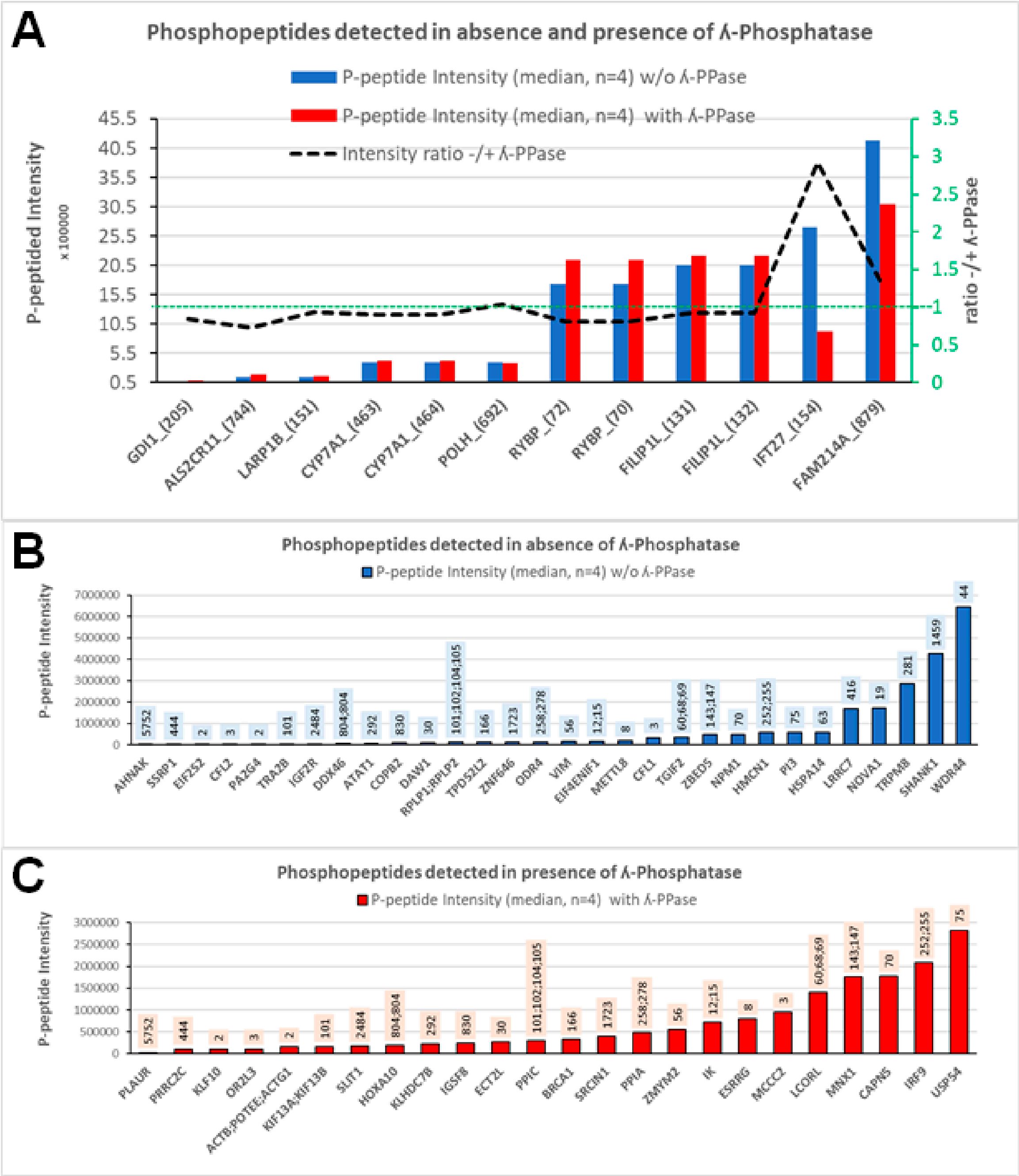

Figure S4. Phosphopeptides and inferred phosphoproteins identified by direct shotgun proteomics (without specific enrichment). **(A)** Phosphopeptide site-intensity-ratios of detected, localized (p>75%) and quantified phosphopeptids (P(STY)) on corresponding proteins (sites in parenthesis) in shotgun measurements of crude or ʎ-Phosphatase (ʎ-PPase) treated DU145-IS-lysates without prior phosphopeptide enrichment (direct shotgun) by the MaxQuant-Andromeda algorithm. **(B, C)** Phosphopeptide site-intensities detected only without **(B)** or with **(C)** ʎ-PPase treatment. Inferred phosphosites in respective proteins are oulined on top of respective bars. Data are obtained from MaxQuant phospho(STY).txt and proteingroups.txt tables and are summarized in Table S4, Sheet 03.

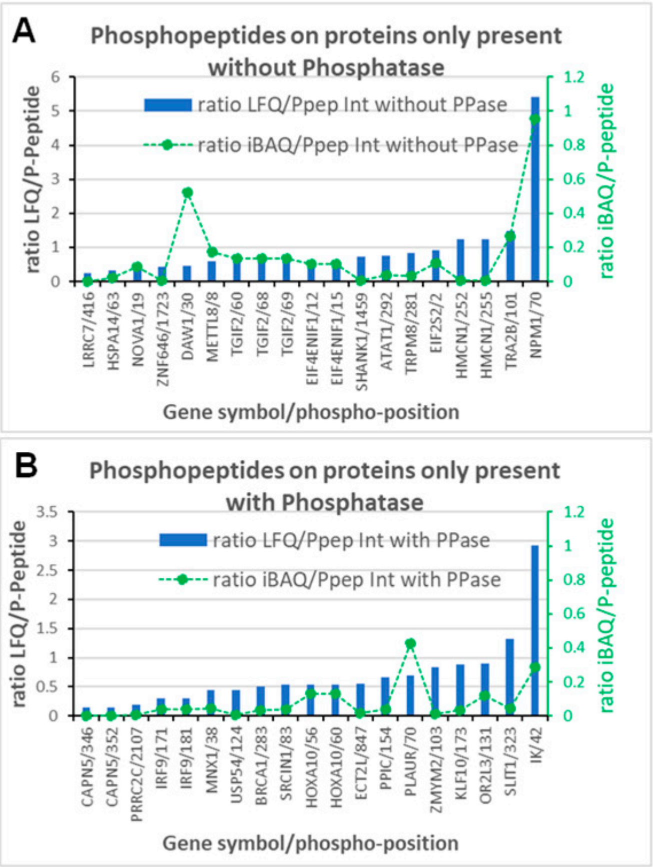

Figure S5. Evaluation of the relative contribution of a phosphopeptide-intensity on the total protein abundance (LFQ) and iBAQ. The MaxQuant algorithm enables to quantify the relative abundance of a protein in a complex peptide mixture by assembling and adding up individual, protein/proteoform-specific peptide intensities into a protein-abundance value, termed LFQ intensity. Importantly, for modification specific peptides, such as phosphopeptides, individual peptide intensities are reported and ratios of modified/un-modified peptides are only reported if the corresponding un-modified peptide is detected- which is not always the case. To evaluate the relative contribution of a phosphopeptide-intensity on the LFQ, we show that the modified peptide intensity contributes to variable degrees and for some proteins substantially to the LFQ protein abundance (HMCN1, TRA28, NPM1 and SLIT1, IK in samples without (shown in **A**) and with ʎ-PPase-treatment, respectively, shown in **B**). In contrast, quantification on the basis of iBAQ intensity (the sum of a protein´s measured peptide intensities are divided by the number of theoretically measureable tryptic peptides) reflects the abundance of the phosphorylated versus the unphosphorylated protein much better as iBAQ/p-peptide ratios mostly lower than 1 in samples without and with phosphatase treatment (green dotted line), with one striking exception-nucleophosmin 1/NPM1. This 294AA long protein has 38 trypsin-cleavage sites, theoretically 12 iBAQ peptides of which we detected 8 and obviously the phosphopeptide contributed significantly to the overall iBAQ ratio.
